# Impact of Nonalcoholic Steatohepatitis on the Outcome of Patients Undergoing Roux-en-Y Gastric Bypass Surgery: a Propensity Score–Matched Analysis

**DOI:** 10.1007/s11695-021-05642-0

**Published:** 2021-09-21

**Authors:** Ziad Abbassi, Lorenzo Orci, Jeremy Meyer, Sebastian Douglas Sgardello, Nicolas Goossens, Laura Rubbia-Brandt, Laurent Spahr, Nicolas Christian Buchs, Stefan Paul Mönig, Christian Toso, Monika Elisabeth Hagen, Minoa Karin Jung

**Affiliations:** 1 Division of Visceral Surgery, Department of Surgery, Geneva University Hospitals and Faculty of Medicine, Rue Gabrielle-Perret-Gentil 4, 1211 Geneva 14, Switzerland; 2 Division of Gastroenterology and Hepatology, Department of Medicine, Geneva University Hospitals and Faculty of Medicine, Geneva, Switzerland; 3 Department of Pathology and Immunology, University of Geneva, Geneva, Switzerland; 4 Division of Clinical Pathology, Geneva University Hospitals, Geneva, Switzerland

**Keywords:** Non-alcoholic fatty liver disease, Non-alcoholic steatohepatitis, Outcome, Roux-en-Y gastric bypass, Propensity score matching, Bariatric surgery

## Abstract

**Purpose:**

It is currently unknown whether NASH (nonalcoholic steatohepatitis), as compared to simple steatosis, is associated with impaired postoperative weight loss and metabolic outcomes after RYGB surgery. To compare the effectiveness of Roux-en-Y gastric bypass (RYGB) on patients with NASH versus those with simple nonalcoholic fatty liver (NAFL).

**Materials and Methods:**

We retrospectively retrieved data from 515 patients undergoing RYGB surgery with concomitant liver biopsy. Clinical follow-up and metabolic assessment were performed prior to surgery and 12 months after surgery. We used multivariate analysis of variance (MANOVA) and propensity score matching and we assessed for changes in markers of hepatocellular injury and metabolic outcomes.

**Results:**

There were 421 patients with simple NAFL, and 94 with NASH. Baseline alanine and aspartate aminotransferases were significantly higher in patients with NASH (*p* < 0.01). Twelve months after the RYGB surgery, as determined by both MANOVA and propensity score matching, patients with NASH exhibited a significantly greater reduction in alanine aminotransferase (*ß*-coefficient − 12 iU/l [− 22 to − 1.83], 95% CI, adjusted *p* = 0.021) compared to their NAFL counterparts (31 matched patients in each group with no loss to follow-up at 12 months). Excess weight loss was similar in both groups (*ß*-coefficient 4.54% [− 3.12 to 12.21], 95% CI, adjusted *p* = 0.244). Change in BMI was comparable in both groups (− 14 (− 16.6 to − 12.5) versus − 14.3 (− 17.3 to − 11.9), *p* = 0.784).

**Conclusion:**

After RYGB surgery, patients with NASH experience a greater reduction in markers for hepatocellular injury and similar weight loss compared to patients with simple steatosis.

**Supplementary Information:**

The online version contains supplementary material available at 10.1007/s11695-021-05642-0.

## Introduction/Purpose

Nonalcoholic fatty liver disease (NAFLD) is the hepatic manifestation of the metabolic syndrome and encompasses a spectrum of clinicopathologic abnormalities that includes nonalcoholic fatty liver (NAFL or simple steatosis), nonalcoholic steatohepatitis (NASH), and liver cirrhosis and its complications [1, 2]. Nonalcoholic fatty liver disease is a leading cause of the global burden of chronic liver disease, affecting up to a quarter of the general adult population, both in high- and low-income countries [3]. Eighty to 90% of obese people display NAFLD and it is estimated that 10–15% of these cases will eventually progress to liver fibrosis and cirrhosis [4]. On top of liver-specific morbidity and mortality, patients with NAFLD are at risk of coronary artery disease and type II diabetes, as well as various types of cancer [5, 6]. In the USA, models designed to predict the public health impact of NAFLD suggest that the number of cases may increase by 21%, from 83.1 million in 2015 to 100.9 million in 2030 [7]. NAFL and NASH are both reversible conditions. In this regard, their alarming epidemiological rise emphasizes the need for the public health community to increase awareness and diagnosis of these conditions and to develop effective interventions.

In patients with morbid obesity, when exercise and diet interventions have failed, bariatric surgery may be indicated. While the benefits and risks of such interventions are well reported [8], there is only scarce evidence as to whether patients within distinct severity stages of NAFLD may experience different weight loss after Roux-en-Y gastric bypass (RYGB) surgery. Furthermore, it is currently unknown whether patients with more severe forms of NAFLD may expect the same benefit from RYGB as those with simple obesity-related fatty liver infiltration.

Such uncertainty is highlighted by the fact that some studies have reported that the severity of steatosis can increase after procedures such as jejunoileal bypass surgery [9]. Conflicting evidence suggests that rapid weight loss can either lead to a reduction in the degree of fatty liver infiltration, or may promote steatohepatitis [10]. While it is generally accepted that bariatric surgery is beneficial to patients with NAFLD, there is a lack of evidence comparing the outcome of RYGB in patients with simple steatosis and those with active NASH. Based on these observations, the primary aim of this study was to compare the effects of RYGB surgery in a large cohort of patients with either simple NAFL or established NASH.

## Materials and Methods

### Patients Selection and Data Collection

This study was approved by the Institutional Review Board (number 2019–00,318) and informed consent was waived. We retrospectively collected information on all patients undergoing RYGB with a concomitant liver biopsy at our center, between January 1997 and December 2013. Patients were divided in two groups: one comprising patients with simple liver steatosis and the other made up of patients with histologically proven NASH (i.e., with lobular inflammation).

We retrieved the following information: patients demographics, American Association of Anesthesiologists (ASA) score, body mass index (BMI), levels of aspartate and alanine aminotransferases (AST, ALT) in the blood, fasting glycemia (mmol/l), fasting insulinemia (µU/m), and obesity-related complications such as presence of type II diabetes (defined as fasting glycemia > 7 mmol/l or HbA1C > 6.5% or under antidiabetic medication) and elevated blood pressure (defined as blood pressure > 140/90 mmHg or medication). Insulin resistance was determined through the homeostatic model assessment for insulin resistance (Homa-IR) [11]. The histological diagnosis of NASH was established based on the steatosis, activity, and fibrosis (SAF) score [12], as determined by a blind histological review of all slides by a senior hepatopathologist. Briefly, the SAF score attributes points according to the grade of liver steatosis, the degree of lobular inflammation, and the extension of fibrosis, while the severity of lobular inflammation determines whether steatohepatitis is present. Excess weight loss (EWL) was calculated as the percent postoperative reduction in body weight, based on an ideal BMI of 25 kg/m^2^.

Clinical and biochemical outcomes were collected at baseline (before surgery) and at the 12-month follow-up visit.

### Surgical Technique

The surgical technique was standardized and similar between the groups. A detailed description of the procedure is available elsewhere [13]. Briefly, a pneumoperitoneum was created using the Optiview (Endopath Xcel, Ethicon) technique, and a 20–30-cm^3^ gastric pouch was constructed using blue or green cartridge staplers as clinically indicated. Next, a standard RYGB with a 150-cm alimentary limb and a 75-cm biliopancreatic limb was tailored. In the laparoscopic approach, a mechanical circular gastrojejunal (GJ) anastomosis with a transorally inserted anvil or a linear GJ anastomosis was carried out, and a jejunojejunal (JJ) anastomosis was performed with a linear stapler. In the robotic approach, hand-sewn GJ and JJ anastomoses were carried out. A routine air and methylene blue leak test was performed at the end of the procedure.

### Statistical Analysis

Because most of the assessed variables did not have a normal distribution, continuous outcomes were evaluated by calculating the median and interquartile range (IQR) and their comparison was performed with the Mann–Whitney *U* test. The primary outcome of interest was the change in the ALT level in the blood, from baseline to 12 months after the RYGB surgery. Secondary outcomes of interest included EWL (%) and changes in AST, fasting insulinemia, and Homa-IR. We first crudely compared the median change of these outcomes from baseline in NASH and NAFL patients, by using the Mann–Whitney *U* test. Next, to further enhance the comparability of patients in the NAFL or the NASH group, we carried out a multivariable analysis of variance (MANOVA), adjusting for the following confounders: patient age, gender, baseline BMI, presence of type II diabetes, and ASA score. Finally, we included an additional approach, by using a logistic regression model to generate propensity scores, taking into account the same aforementioned confounders. The nearest matching neighbor algorithm was then used to achieve 1:1 propensity score matching. Statistical analyses were performed using SPSS (Version 22, SPSS Inc., Chicago, Illinois) and STATA (version 12, Stata Corp, College Station, Texas). All authors had access to the study data and reviewed and approved the final manuscript.

## Results

**Fig. 1:**
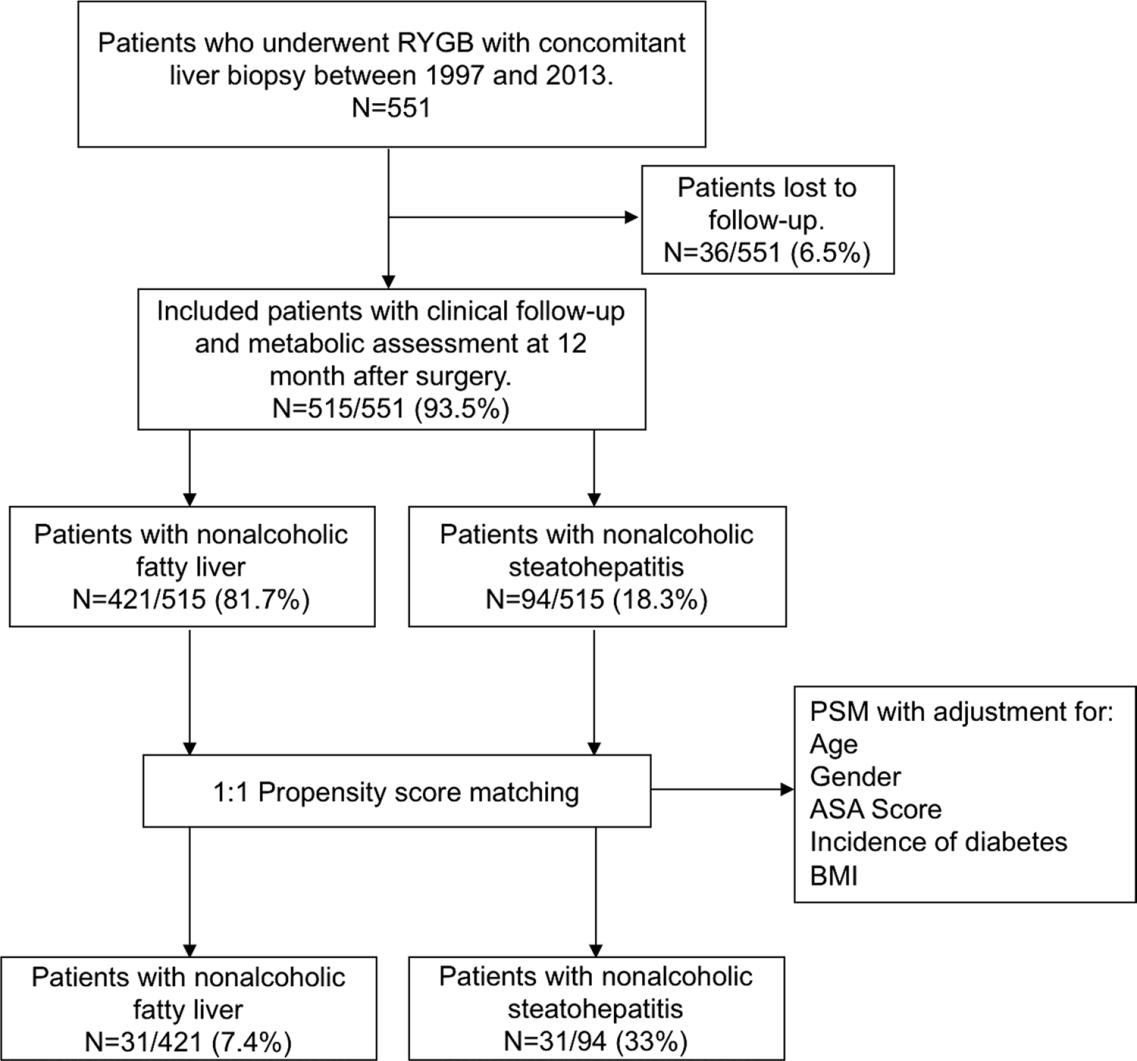
Flow chart. RYGB, Roux-en-Y gastric bypass; PSM, propensity score matching; ASA, American Society of Anaesthesiologist; BMI, body mass index

During the period covered by our study, 551 patients underwent RYGB surgery with concomitant liver biopsy at our center. One year after surgery, 6.5% of patients were lost to follow-up; therefore, we analyzed data collected on 515 patients (Fig. 1). Based on the SAF score, there were 421 patients with liver steatosis (NAFL group) and 94 with active lobular inflammation (NASH group). Baseline clinical characteristics are shown in Table 1. Patients in the NASH group were more commonly males, had higher glycemia, Homa-IR values, and levels of aminotransferases in the blood, and presented a more severe grade of liver steatosis. There was also a higher prevalence of type II diabetes and hypertension in the NASH group.

**Table 1:** Baseline characteristics and after propensity score matching

	Patients with NAFL entire cohort (*n* = 421)	Patients with NASH entire cohort (*n* = 94)	*p* value	Patients with NAFL after PSM (*n* = 31)	Patients with NASH after PSM (*n* = 31)	*p* value
Age (years), IQR	40 (33–49)	45 (39–52)	0.0004	46 (37–54)	45 (40–52)	0.9494
Gender female, *n* (%)	350 (83%)	52 (55%)	< 0.0001 *	18 (58%)	18 (58%)	1.0000 *
ASA score, IQR	2 (2–3)	3 (2–3)	< 0.0001	2 (2–3)	3(2–3)	0.8011
BMI (kg/m^2^), IQR	43.3 (41–47)	44.5 (41–49)	0.0436	44.7 (42–48)	43.6 (41.4–49)	0.6573
AST (iU/l), IQR	19 (15–25)	30 (24–41)	< 0.0001	18 (15–24)	30 (25–41)	0.0001
ALT (iU/l), IQR	25 (17–37)	47 (38–70)	< 0.0001	25.5 (16–38)	53 (41–70)	< 0.0001
Liver steatosis (%), IQR	30 (10–60)	70 (60–80)	< 0.0001	40 (15–60)	72.5 (60–90)	< 0.0001
Glycemia (mmol/l), IQR	5.8 (5.2–6.6)	6.8 (5.8–9)	< 0.0001	6.2 (5.6–7)	6.6 (6.1–10.4)	0.0732
Insulinemia (miU/l), IQR	19.7 (14–30)	28 (19.4–43.6)	< 0.0001	20.5 (17.2–28.5)	32.5 (19.2–62.5)	0.0212
Homa-IR, IQR	5.5 (3.5–8.7)	9.3 (5.9–16.3)	0.0031	3.6 (2.6–5.5)	4.4 (2.8–8.4)	0.3514
Type II diabetes, *n* (%)	98 (23%)	45 (48%)	< 0.0001 *	13 (42%)	11 (36%)	0.2719 *
Hypertension, *n* (%)	139 (33%)	51 (54%)	< 0.0001 *	15 (48%)	13 (42%)	0.2605 *
Triglycerides (mmol/l), IQR	1.3 (0.9–2)	1.8 (1.2–2.4)	< 0.0001	1.4 (1.2–2.1)	1.8 (1.22–2.11)	0.2308

Data show median and interquartile range (IQR), unless otherwise specified

Abbreviations: *NAFL*, nonalcoholic fatty liver; *NASH*, nonalcoholic steatohepatitis; *ALT*, alanine aminotransferase; *AST*, aspartate aminotransferase; *ASA*, American Association of Anesthesiologists; *BMI*, body mass index; *Homa-IR*, homeostatic model assessment for insulin resistance. Data were compared using the Mann–Whitney *U* test, unless specified otherwise. *Chi-squared test

We first assessed outcomes in the whole cohort, comparing between-group changes from baseline to 12 months after the RYGB surgery. In the entire cohort, 3 patients in the NAFL group and 22 patients in the NASH group were lost to follow-up at 12 months. As shown in Table 2, we found that patients in the NASH group experienced a significantly greater reduction in ALT (*p* < 0.001), AST (*p* < 0.001), glycemia (*p* < 0.001), fasting insulinemia (*p* = 0.014), and Homa-IR (*p* = 0.001). At the end of follow-up, the NASH group still had a higher proportion of patients with type II diabetes compared to the NAFL group (9% vs. 1.5%, *p* = 0.001). In this unadjusted analysis, patients in the NASH group displayed a less marked EWL compared to the patients in the NAFL group. The EWL was 71.9% (55.9–89.3%) in the NASH group vs. 77.3% (65.3–90.5%) in the NAFL group, *p* = 0.012. Median change in BMI at 12 months was comparable in both groups (− 14.9 (− 17 to − 12.3) vs − 14.6 (− 16.7 to − 11.45), *p* = 0.448).

**Table 2:** Outcomes in the whole cohort

	Patients with NAFL (*n* = 421)			Patients with NASH (*n* = 94)			
	Baseline (*n* = 421)	12 months (*n* = 418)	Median change from baseline	Baseline (*n* = 94)	12 months (*n* = 69)	Median change from baseline	*p* value*
ALT(iU/l)	25 (17 to 37)	21 (15 to 28)	− 4 (− 14 to 5)	47 (38 to 70)	26 (20 to 34)	− 22 (− 41 to − 10)	< 0.001
AST (iU/l)	19 (15 to 25)	21 (17 to 26)	2.5 (− 4 to 8)	30 (24 to 41)	23 (20 to 30)	− 5.5 (− 15.5 to 4)	< 0.001
EWL (%)	-	77.3 (65.3 to 90.5)	-	-	71.9 (55.9 to 83.3)	-	0.012
BMI (kg/m^2^)	43.3 (41 to 47)	29.2 (26.6 to 32.2)	− 14.9 (− 17 to -12.3)	44.5 (41 to 49)	29.9 (27.8 to 36)	− 14.6 (− 16.7 to − 11.45)	0.448
Glycemia (mmol/l)	5.8 (5.2 to 6.6)	4.78 (4.4 to 5.2)	− 1.02 (− 2 to − 0.5)	6.75 (5.8 to 9)	4.98 (4.6 to 5.6)	− 1.73 (− 2.87 to − 0.9)	< 0.001
Insulinemia (μU/L)	19.7 (14 to 30)	6.5 (4.75 to 8.45)	− 13.4 (− 22 to − 7.9)	28 (19.4 to 43.6)	9.1 (7.5 to 14)	− 22.5 (− 52.9 to − 10.3)	0.014
Homa-IR	5.46 (3.48 to 8.56)	1.38 (0.97 to 1.95)	− 3.87 (− 6.62 to − 2)	9.27 (5.93 to 16.32)	2.01 (1.52 to 3.01)	− 8.92 (− 19.8 to − 3.18)	0.001

Data show median and interquartile range, unless otherwise specified

Abbreviations: *NAFL*, nonalcoholic fatty liver; *NASH*, nonalcoholic steatohepatitis; *AST*, aspartate aminotransferase; *ALT*, alanine aminotransferase; *EWL*, excess weight loss; *BMI*, body mass index; *Homa-IR*, homeostatic model assessment for insulin resistance

^*^p value for the difference in median change from baseline

Next, we carried out a MANOVA test, adjusting for patient age, gender, baseline BMI, presence of type II diabetes, and ASA score (Table 3). Using this approach, we found that, compared to patients with simple steatosis, patients with NASH experienced a significantly greater adjusted decrease in ALT (*β* coefficient − 12 iU/l [− 22 to − 1.83], *p* = 0.021), insulinemia (*β* coefficient − 10.76 mIU/l [− 17.01 to − 4.51], *p* = 0.001), and Homa-IR (*β* coefficient − 5.01 [− 7.06 to − 2.95], *p* < 0.001). In contrast, the adjusted impact of NASH on the reduction in AST (*β* coefficient − 1.81 iU/l [− 8.98 to 5.36], *p* = 0.619) and on EWL (*β* coefficient 4.54% [− 3.12 to 12.21], *p* = 0.244) was no longer significant.

**Table 3:** Multiple analysis of variance, adjusted for age, gender, body mass index, type II diabetes, and ASA

	Alanine aminotransferase		Aspartate aminotransferase		Insulinemia		Homa-IR		Excess weight loss	
	Coeff	*p* value	Coeff	*p* value	Coeff	*p* value	Coeff	*p* value	Coeff	*p* value
Nonalcoholic steatohepatitis (versus NAFL)	− 12 (− 22 to − 1.83)	0.021	− 1.81 (− 8.98 to 5.36)	0.619	− 10.76 (− 17.01 to − 4.51)	0.001	− 5.01 (− 7.06 to − 2.95)	< 0.001	4.54 (− 3.12 to 12.21)	0.244

Data indicate change from baseline to 12 months post Roux-en-Y gastric bypass surgery. Coefficients of the multiple analysis of variance go along with 95% confidence intervals

Abbreviations: *Homa-IR*, homeostatic model assessment for insulin resistance; *NAFL*, nonalcoholic fatty liver

In another attempt to allow for confounding variables, we constructed propensity scores, adjusting for patient age, gender, baseline BMI, presence of type II diabetes, and ASA score. Using this approach, we identified *n* = 31 pairs of NASH and NAFL patients that were otherwise comparable in terms of the aforementioned confounders. We reassessed changes from baseline to 12 months post-RYGB surgery between these matched patients; no patient was lost to follow-up at 12 months (Table 4). Consistent with the MANOVA test, the results of the propensity score matching analysis indicated that patients in the NASH group displayed a significant reduction of ALT levels in the blood compared to patients with simple steatosis with a median change from baseline of − 25iU/l (− 38 to − 16) in the NASH group vs. − 5 iU/l (− 21 to 4) in the NAFL group, *p* = 0.003. Both groups experienced similar EWL and change in BMI. The results of the propensity score matching analysis further indicated a significant difference in terms of change in AST with a median change from baseline of − 7 iU/l (− 15 to − 3) in the NASH group vs. 3 iU/l (− 3 to 8) in the NAFL group, *p* = 0.007. There was a trend of borderline significance supporting a more marked reduction in fasting insulinemia in patients with NASH (*p* = 0.064), while Homa-IR changes were not significantly different between groups.

**Table 4:** Outcome assessment after propensity score matching

	NAFL patients (*n* = 31)			NASH patients (*n* = 31)			
	Baseline (*n* = 31)	12 months (*n* = 31)	Change from Baseline	Baseline (*n* = 31)	12 months (*n* = 31)	Change from baseline	*p* value for the difference in median change from baseline
ALT (iU/l)	25.5 (16 to 38)	19 (15 to 26)	− 5 (− 21 to 4)	53 (41 to 70)	24 (20 to 32)	− 25 (− 38 to − 16)	0.003
AST (iU/l)	18 (15 to 24)	23 (17 to 27)	3 (− 3 to 8)	30 (25 to 41)	23 (20 to 30)	− 7 (− 15 to − 3)	0.007
EWL (%)	-	72.3 (65.9 to 78.6)	-	-	74.4 (68.1 to 80.7)		0.598
BMI change (kg/m^2^)	44.7 (42 to 48)	31.5 (27.5 to 34.7)	− 14 (− 16.6 to − 12.5)	43.6 (41.4 to 49)	29.8 (27.6 to 33.1)	− 14.3 (− 17.3 to − 11.9)	0.784
Insulinemia	20.5 (17.2 to 28.5)	6.3 (4.9 to 8.6)	− 14.5 (− 18 to − 10.5)	32.5 (19.2 to 62.5)	9.1 (7.5 to 14)	− 24.8 (− 57.3 to − 8.5)	0.064
Homa-IR	3.60 (2.58 to 5.50)	1.39 (1.04 to 2.18)	− 2.14 (− 3.37 to − 0.56)	4.4 (2.81 to 8.44)	2.14 (1.55 to 3.03)	− 1.76 (− 5.22 to − 1.022)	0.597

Data show median ± interquartile range

Abbreviations: *NAFL*, nonalcoholic fatty liver; *NASH*, nonalcoholic steatohepatitis; *ALT*, alanine aminotransferase; *AST*, aspartate aminotransferase; *EWL*, excess weight loss; *Homa-IR*, homeostatic model assessment for insulin resistance

## Discussion

The current study provides a head-to-head comparison of the impact of RYGB in patients with simple steatosis versus those with active steatohepatitis. The present study uses two different statistical approaches to adjust for baseline imbalance between groups, and results consistently indicate that patients with NASH experience similar excess weight loss as patients with simple steatosis, but a significantly greater reduction in alanine aminotransferase, 12 months post-surgery. The MANOVA test also uncovered some evidence that insulinemia and insulin resistance may decrease to a greater extent in patients with NASH. However, these findings could not be reproduced in the propensity score–matched analysis. Such discrepancy between the two statistical models may be explained either by (a) the presence of a type II error after propensity score matching (due to the limited sample size, *n* = 31 matched pairs) or (b) overmatching. Taking type II diabetes into account in the matching process may have impacted the estimated differences in outcomes such as insulinemia and Homa-IR.

It is worth noting that the patients included in our study did not undergo a second liver biopsy at the end of follow-up. While this may be considered a limitation, one must acknowledge that a liver biopsy is a procedure that comes with potential complications, and that this procedure is not ethically justifiable outside the scope of a dedicated prospective clinical trial. In this regard, there is a large body of evidence documenting that RYGB surgery has a beneficial impact on NAFLD/NASH-associated liver parenchymal abnormalities [14]. A recent meta-analysis indicated that 91% of patients undergoing RYGB experienced a reduction in the histological severity of liver steatosis after surgery [15]. Consistent with these observations, a study by Winder et al. indicated a reduction in steatosis grade in 84% of patients after RYGB surgery [16]. When looking specifically at steatohepatitis, de Almeida et al. reported a complete resolution of necroinflammatory changes and an improvement of liver fibrosis in all NASH patients undergoing RYGB [17]. Similarly, Barker et al. showed that histological NASH features disappear in up to 89% of patients after RYGB surgery [18]. With these optimistic figures in mind, one must acknowledge that not all patients may be fully cured of NAFLD after RYGB [14]. In this regard, putative predictors of a poor improvement in liver histology after RYGB include refractory insulin resistance and limited postoperative weight loss [19, 20]. However, the biological mechanisms explaining the failure of some patients to resolve liver parenchymal abnormalities remain elusive.

The main strength of our study is that the distinction of simple steatosis vs. active NASH was based on liver histology, which remains the gold-standard technique in this area [21, 22]. The histological assessment was done by a senior liver pathologist using the widely validated SAF score [12]. This approach confers robustness to our study by limiting potential misclassification, a source of error that commonly hampers the interpretation of retrospective studies. Moreover, our study, which is based on a large sample size (*n* = 515), is the first to provide a head-to-head comparison of NASH vs. simple steatosis in the context of RYGB surgery. Finally, the current results convey the clinically relevant message that patients with increasingly severe stages of NAFLD should not be prevented from undergoing RYGB, as they may benefit from this intervention to a similar extent as patients with milder forms of NAFLD.

However, our study also has limitations. First, it is based on a retrospectively collected data and therefore, it is by definition prone to some selection bias. For instance, we cannot rule out that dosage and duration of antidiabetic treatment may have differed between study groups, thereby impacting metabolic outcomes of the current study. Second, our follow-up period is relatively short. Hence, one may speculate that weight regain (which commonly occurs beyond 2 years after surgery) could lead to a recurrence of fatty liver infiltration, hepatocellular injury, and liver fibrosis [23, 24]. Third, as mentioned above, we did not repeat the liver biopsy at 12 months, because such a procedure is not recommended in routine care. Therefore, we could not evaluate whether the greater reduction in ALT in the NASH group was actually paralleled by a marked improvement in liver histology. Fourth, one may consider that our propensity score matching incorporates too many adjustment variables and that this may have led to both a marked lowering of the sample size and a likelihood of overmatching. With this limitation in mind, it is worth noting that main result of our study (that is, a greater reduction in ALT in NASH patients and a comparable EWL as compared to those with simple steatosis) was consistently identified both in the MANOVA test and the propensity score matching. Finally, while at the time of inclusion, none of the included patients was known for chronic alcohol intake (as assessed both clinically, and by liver histology), one cannot fully rule out a potential impact of alcohol on postoperative ALT values.

## Conclusion

In summary, the current study compares for the first time the outcomes of patients with NASH versus those with simple steatosis after RYGB surgery. Our results indicate that patients with NASH are appropriate candidates for this surgical procedure, as evidenced by a more profound reduction in markers of hepatocellular injury with similar excess weight loss, as compared to obese counterparts with simple steatosis.

## Supplementary Information

Below is the link to the electronic supplementary material.

Supplementary file1 (XLSX 402 KB)

Supplementary file2 (XLSX 45 KB)
